# Comparison of oseltamivir and α-galactosylceramide for reducing disease and transmission in pigs infected with 2009 H1N1 pandemic influenza virus

**DOI:** 10.3389/fvets.2022.999507

**Published:** 2022-10-20

**Authors:** Darling Melany de C. Madrid, Weihong Gu, Bianca L. Artiaga, Guan Yang, Julia Loeb, Ian K. Hawkins, William L. Castleman, John A. Lednicky, Jürgen A. Richt, John P. Driver

**Affiliations:** ^1^Division of Animal Sciences, University of Missouri, Columbia, MO, United States; ^2^Department of Animal Sciences, University of Florida, Gainesville, FL, United States; ^3^Department of Diagnostic Medicine and Pathobiology, College of Veterinary Medicine, Kansas State University, Manhattan, KS, United States; ^4^Department of Infectious Diseases and Public Health, City University of Hong Kong, Hong Kong, Hong Kong SAR, China; ^5^Department of Environmental and Global Health, University of Florida, Gainesville, FL, United States; ^6^Emerging Pathogens Institute, University of Florida, Gainesville, FL, United States; ^7^Department of Comparative, Diagnostic, and Population Medicine, University of Florida, Gainesville, FL, United States

**Keywords:** α-galactosylceramide, antiviral, invariant natural killer T-cells, oseltamivir, swine, influenza

## Abstract

Influenza virus infections are a major cause of respiratory disease in humans. Neuraminidase inhibitors (NAIs) are the primary antiviral medication used to treat ongoing influenza infections. However, NAIs are not always effective for controlling virus shedding and lung inflammation. Other concerns are the emergence of NAI-resistant virus strains and the risk of side effects, which are occasionally severe. Consequently, additional anti-influenza therapies to replace or combine with NAIs are desirable. Here, we compared the efficacy of the NAI oseltamivir with the invariant natural killer T (iNKT) cell superagonist, α-galactosylceramide (α-GalCer), which induces innate immune responses that inhibit influenza virus replication in mouse models. We show that oseltamivir reduced lung lesions and lowered virus titers in the upper respiratory tract of pigs infected with A/California/04/2009 (CA04) pandemic H1N1pdm09. It also reduced virus transmission to influenza-naïve contact pigs. In contrast, α-GalCer had no impact on virus replication, lung disease, or virus transmission, even when used in combination with oseltamivir. This is significant as iNKT-cell therapy has been studied as an approach for treating humans with influenza.

## Introduction

Influenza infections are a leading cause of morbidity and mortality in humans (1, 2). They also present a substantial burden for swine and poultry producers (3). Moreover, influenza infections of livestock occasionally give rise to zoonotic influenza virus strains that can be transmitted to humans and have the potential to cause human pandemics (4). Vaccination is the primary strategy for controlling influenza infections in humans and livestock. However, influenza vaccines seldom provide long-lasting protective immunity even within the same subtype and there is often insufficient time to produce vaccines against emerging strains (5). Thus, there is a need for antiviral therapies that can reduce influenza-related illness and control the spread of influenza viruses.

Oseltamivir, a neuraminidase inhibitor (NAI) (5), is the most widely prescribed influenza drug in the world and is available as an inexpensive generic medication in many countries. Although there is now strong evidence that oseltamivir shortens the duration of symptoms of influenza-like illness, the extent to which the drug impacts lung pathology, hospitalizations, and mortality due to influenza infections remains controversial (6–10). It has also been reported that oseltamivir may be of limited use for interrupting influenza transmission as oseltamivir treatment does not completely prevent virus shedding (11). Thus, there is a need for additional studies to clarify the efficacy of NAIs for treating human influenza infections.

The current work used pigs to evaluate oseltamivir treatment for controlling influenza infection and disease. Swine are considered a highly-reliable translational model of human influenza infections since (i) they are natural hosts for the same influenza A virus (IAV) subtypes as humans, (ii) they develop clinical disease that parallels human clinical symptoms, (iii) their respiratory tract anatomy and physiology closely resembles that of humans, and (iv) swine anti-influenza immune responses mirror what is observed in humans (12).

In addition to the evaluation of oseltamivir, our study assessed whether influenza virus infections can be mitigated by therapeutically targeting a subset of innate T-cells known as invariant natural killer T-cells (iNKT-cells). iNKT-cells bridge the innate and adaptive immune systems and can stimulate early innate immune responses in barrier organs, including the lungs (13, 14). Unlike conventional T lymphocytes, iNKT-cells express a restricted T-cell receptor (TCR) repertoire which recognize a limited selection of lipid/glycolipid antigens presented by the MHC class-I like CD1d molecule (15). Previous studies in mice have demonstrated that administration of iNKT-cell agonist to IAV-infected mice induced an antiviral immune response that substantially reduced the severity and duration of IAV infections (16–19). While these reports suggest that targeting the immunoregulatory activities of iNKT-cells may be a promising strategy to mitigate influenza infections, uncertainty remains about the clinical translatability of this approach since the average frequency of iNKT-cells in humans is significantly lower than in most inbred mouse strains (20). In the current work, our goal was to determine if iNKT-cell therapy, alone or in combination with oseltamivir, is a feasible approach to treat influenza virus infections in pigs as this species is similar to humans in regard to iNKT-cells and anti-influenza immune responses (12, 21).

## Materials and methods

### Pigs

Commercial mixed-breed pigs were obtained from the University of Florida Swine Unit and Midwest Research Swine (Gibbon, MN). The experiments were performed in compliance with guidelines from the United States Department of Agriculture and the National Research Council's Guide for the Care and Use of Laboratory Animals. The institutional animal care and use committee (IACUC) at the University of Florida approved the protocol under study number 201708209.

### Experimental design

Three-week-old pigs seronegative for antibodies against H1N1, H3N2, and B influenza viruses were assigned to one of five treatment groups. At 4 weeks of age, the pigs were anesthetized with BAM^TM^ combination drug (Butorphanol, Azaperone, Medetomidine) at a dose rate of 1.0 ml per 75 lbs body weight and intratracheally (i.t.) inoculated with 1 x 10^6^ TCID_50_ 2009 pandemic H1N1 A/California/04/2009 (H1N1pdm09) influenza virus, as previously described (22). One group of pigs (αGC group) was intranasally (i.n.) administered 100 μg/kg α-galactosylceramide (α-GalCer) dissolved in 1.5 ml PBS and 2% DMSO, at the time of infection. Another group was orally administered 75 mg oseltamivir phosphate (Lupin Pharmaceuticals) twice a day during the first 5 days after infection (OS group). A third group of pigs received a combination of the α-GalCer and OS treatments (αGCOS group). An additional group of pigs (MC group) was mock treated with 1.5 ml of the α-GalCer vehicle solution (PBS, 2% DMSO), administered i.n. at the time of challenge. Finally, a group of control pigs (MM group) was mock infected with virus-free Dulbecco's Modified Eagle Medium (DMEM) and mock treated with the α-GalCer vehicle solution.

Pigs were monitored daily for clinical disease as previously described (23). To quantify virus shedding, nasal swabs were collected daily from −1 to 5 days post infection (d.p.i.). Blood samples were collected on −1, 3, and 5 d.p.i. for flow cytometry. Pigs were euthanized 5 days after infection and tissue samples from the lung, trachea, bronchus, nasal turbinates, spleen, and bronchoalveolar lavage fluid (BALF) were collected and analyzed by virus titration, flow cytometry, and RT-qPCR, as previously described (24). The right middle lung lobe was collected for histopathology.

In another study, 16 four-week-old pigs were divided among three groups that were treated identically to the MC, OS, and αGC groups in the first study, except that 2 days after virus challenge five or six IAV-naïve contact pigs were co-housed with each group for 3 days. Additionally, BioSampler^®^ air sampling devices (SKN, Inc.) were placed 50 cm away from two of the pens housing MC and OS groups of pigs at 3 d.p.i. The samplers collected 720 liters of air over 90 min into 15 ml of PBS, which was reduced to 400 μl by centrifugation. Primary inoculated and contact pigs were necropsied at 5 d.p.i. and 5 days post contact (d.p.c.), respectively. Necropsies were performed identically to the first experiment.

### Flow cytometry

Lung and spleen samples were dispersed into single cells as previously described (23). Blood and tissue samples were treated with an ammonium chloride-based erythrocyte lysis buffer (23). Cells were blocked with polyclonal rat IgG Ab, stained with tetramer and antibody reagents, and acquired using Attune NxT flow cytometer (Thermo Fisher Scientific). Reagents used to analyze iNKT-cells, αβ and γδ T-cells, monocytes, macrophages, and granulocytes are described in Supplemental Table 1. Data were analyzed using FlowJo software (V 10.6.1, BD Biosciences).

### Virus and viral titers

Influenza virus encoding the original consensus sequence of the H1N1pdm09 strain A/California/04/2009 (CA04) was generated by reverse genetics as previously described (25). Viral titers were calculated as the median TCID_50_, and viral titers expressed as Log_10_ TCID_50_/ml or Log_10_ TCID_50_/g as appropriate. TCID_50_ values were determined as previously described (24).

### Lung immunopathology

The right middle lung lobe was perfused with 10% neutral-buffered formalin. Four blocks were sampled and embedded in paraffin. Seven μm-thick paraffin sections were stained with hematoxylin and eosin (H&E) for histopathologic analysis. The severity of bronchitis and bronchiolitis was scored according to the size of leukocyte aggregations in bronchial and bronchiolar walls, respectively. Each section was scored from 0 to 3 where a score of 0 represents no lesions; a score of 1 represents a low leukocyte density in airway walls (i.e., bronchus or bronchiole); a score of 2 represents a moderate leukocyte density in airway walls; and a score of 3 represents a high leukocyte density wrapping around the entire circumference of the airway wall. Pneumonia severity was scored according to the size of intra-alveolar lesions as follows: 0: no lesions; a score of 1 represents small areas of thickened interalveolar septa, type 2 pneumocyte hyperplasia, leukocyte septal infiltration, and low densities of intra-alveolar leukocytes; a score of 2 represents moderately-sized areas of thickened interalveolar septa, type 2 pneumocyte hyperplasia, leukocyte septal infiltration, and moderate densities of intra-alveolar leukocytes; a score of 3 represents large areas of thickened interalveolar septa, type 2 pneumocyte hyperplasia, leukocyte septal infiltration, and high densities of intra-alveolar leukocytes. The prevalence of bronchitis, bronchiolitis, and pneumonia lesions was scored from 0 to 3 as follows: (0): no lesions, (1): focal to multifocal lesions, (2): locally extensive lesions (between 20 and 45% of the section or airways), (3): diffuse or diffusely multifocal lesions throughout the section (representing >45% of tissue section area). Pneumonia, bronchitis, and bronchiolitis scores were summed to calculate an overall severity score out of a maximum possible score of 12. An overall disease prevalence score was calculated by multiplying the overall severity score by the prevalence score, with a maximum possible score of 36.

### RNA isolation and RT-qPCR

Cranial, middle, and caudal tissue lung samples (3 mg of each) were combined and homogenized using a Precellys homogenizer (Bertin). RNA was isolated using QIAzol Lysis Reagent (Qiagen, 79306) with the RNeasy Lipid Tissue mini kit (Qiagen, 74804) and reverse transcribed to cDNA using a Superscript VILO Master Mix (Thermo Fisher Scientific, 11755-050). cDNA was mixed with a Fast SYBR Green Master Mix (Applied Biosystems, 43-856-12). The PCR mixture was dispensed into a quantitative reverse transcriptase PCR (RT-qPCR) array (PASS-011ZF, Qiagen), which profiles 84 inflammatory cytokine/chemokines genes. The RT-qPCR reaction was carried out using a CFX Connect real-time cycler (Bio-Rad) according to the manufacturer's recommended cycling conditions. Gene expression was quantified according to standard ΔΔCt methods using the geometric mean of three housekeeping genes (*ACTG1, B2M, RPL13A*) that are included in each array. A two-tailed Student's *t*-test was used to compare gene expression between treatments. Differentially expressed genes were subjected to a Kyoto Encyclopedia of Genes and Genomes (KEGG) pathway enrichment analysis using Database Annotation, Visualization and Integrated Discovery (DAVID) version 2021 (https://david.ncifcrf.gov/). Heatmaps were generated in GraphPad Prism using the geometric mean of the gene expression of three to six samples per treatment.

### Statistical analysis

Data was graphed and analyzed using GraphPad Prism, version 9.1.0 (GraphPad Software). TCID_50_ data were log transformed to address the heteroscedasticity and non-normally distributed residuals of untransformed data and analyzed using a mixed-effect model. Means were separated using Turkey's test when a main effect or interaction term was significant (*P* < 0.05). A Fisher's exact test was used to compare the proportion of infected animals between treatment groups and a Log-rank test was used to compare viral infection incidence curves. Welch's *t*-test was used to compare virus titers in respiratory tissues due to their unequal variances among treatment groups. Pathology scores were assessed using a one-way analysis of variance (ANOVA) followed by the Mann-Whitney *U* test. Flow cytometric data were analyzed on RStudio version 1.4.1103 using glm function followed by pairwise comparisons with Tukey's test.

## Results

### Effect of oseltamivir and α-GalCer on virus shedding and replication

**Figure 1:**
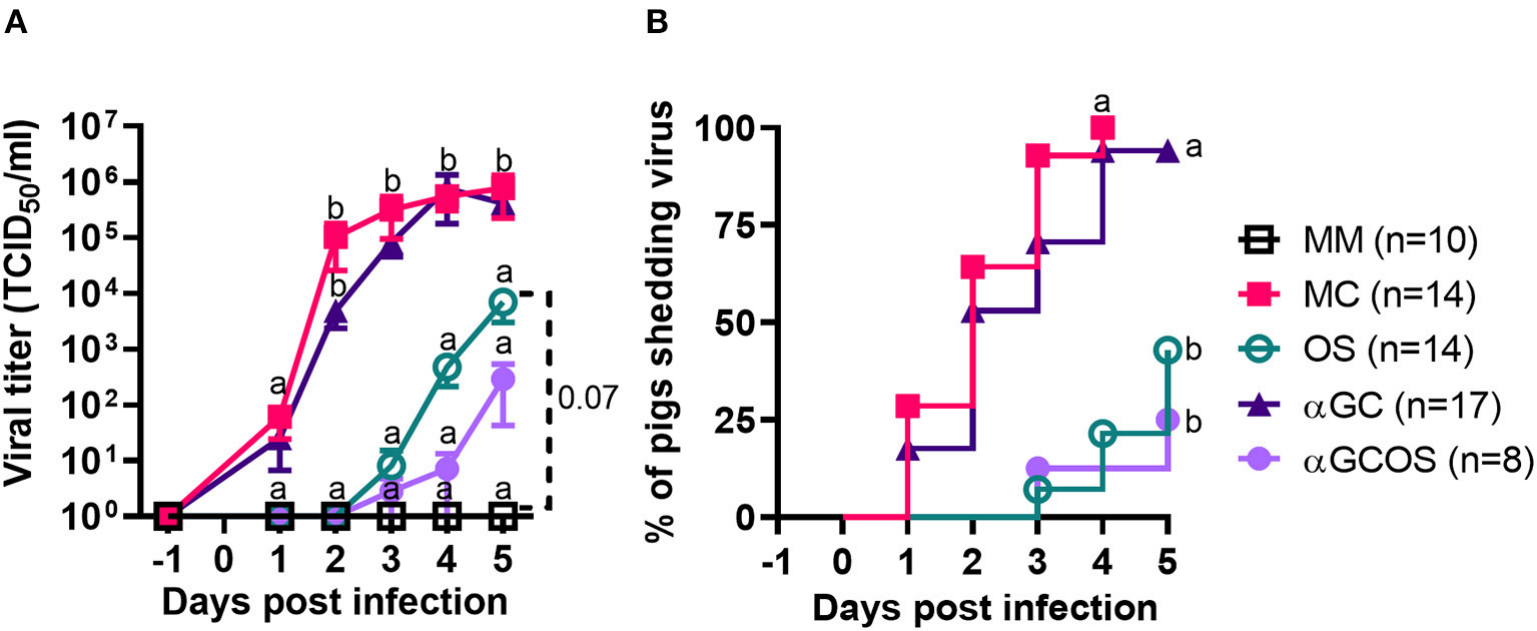
Virus levels in nasal secretions. **(A)** Virus titers in nasal swabs collected at −1 to 5 d.p.i. from pigs inoculated with H1N1pdm09. Data are represented as mean ± standard error of the mean (SEM). Values within days with no common superscript letters differ significantly (*P* < 0.05) when analyzed using Tukey's test. Values with the same superscript are not statistically different. **(B)** Incidence of pigs shedding virus. Survival curves with no common superscript letters differ significantly (*P* < 0.05) when compared by the log-rank test. Survival curves with the same superscript are not statistically different.

Virus shedding was significantly delayed and reduced in oseltamivir treated pigs (OS group) compared to mock treated pigs (MC group) (Figure 1). Virus positive nasal swabs were detected in 3/14 OS and 14/14 MC pigs at 4 d.p.i. when oseltamivir treatment was discontinued. An additional 3 OS pigs started shedding virus on 5 d.p.i. Shedding was similar between MC pigs and pigs treated with α-GalCer monotherapy (αGC group). Treating pigs with the combination of α-GalCer and oseltamivir (αGCOS group) did not significantly reduce the proportion of virus-shedding pigs compared to pigs treated with oseltamivir alone. However, αGCOS pigs tended to shed less virus than OS pigs at 4 and 5 d.p.i.

Analysis of virus titers in the respiratory tissues at 5 d.p.i. found that OS pigs had reduced titers in nasal turbinates, trachea, and bronchi compared to MC pigs (Table 1). They also tended to have lower titers in BALF and lung tissue. Oseltamivir treatment decreased the proportion of pigs with detectable levels of virus in nasal turbinates, trachea, and BALF. The effect was less striking for lung samples as fewer MC lung samples had detectable levels of virus at 5 d.p.i. compared to the other tissues. α-GalCer monotherapy did not significantly reduce virus titers or the proportion of virus positive samples for any of the tissues tested. However, we did observe that αGC pigs tended to have lower virus titers in caudal lung samples compared to the MC pigs. αGCOS pigs were similar to OS pigs for virus titers and the proportion of virus positive samples.

**Table 1:** Virus titers of BALF and homogenized respiratory tissues at 5 d.p.i.

**Tissue**	**MM**	**MC**	**OS**	**αGC**	**αGCOS**
BALF	0/8 (0.00 ± 0.00)^a^	**11/12 (2.39** **±0.31)**^**b**^	**4/11 (1.27** **±0.55)**^**b**^	**11/14 (2.16** **±0.36)**^**b**^	**5/8 (1.34** **±0.52)**^**b**^
Turbinate	0/8 (0.00 ± 0.00)^a^	**7/11 (3.44** **±0.81)**^**b**^	2/11 (0.75 ± 0.51)^ac^	**12/14 (4.27** **±0.60)**^**b**^	0/7 (0.00 ± 0.55)^ac^
Trachea	0/8 (0.00 ± 0.00)^a^	**11/12 (5.04** **±0.48)**^**b**^	**5/11 (2.36** **±0.85)**^**c**^	**12/14 (4.64** **±0.55)**^**bd**^	**5/8 (2.72** **±0.81)**^**cd**^
Bronchus	0/8 (0.00 ± 0.00)^a^	**8/11 (3.78** **±0.80)**^**b**^	**4/11 (1.23** **±0.53)**^**c**^	**9/14 (3.54** **±0.65)**^**b**^	2/8 (1.17 ± 0.80)^ac^
Cranial	0/8 (0.00 ± 0.00)^a^	3/12 (1.96 ± 1.00)^a^	4/11 (1.95 ± 0.83)^a^	5/14 (1.64 ± 0.62)^a^	1/8 (0.60 ± 0.6)^a^
Middle	0/8 (0.00 ± 0.00)^a^	**5/12 (2.27** **±0.87)**^**bc**^	2/11 (0.89 ± 0.60)^ab^	**10/14 (3.90** **±0.76)**^**c**^	1/8 (0.61 ± 0.61)^ab^
Caudal	0/8 (0.00 ± 0.00)^a^	6/12 (2.10 ± 0.70)^b^	3/11 (1.05 ± 0.66)^ab^	2/14 (0.57 ± 0.39)^ab^	1/8 (0.61 ± 0.74)^ab^

Results are shown as the number of pigs positive for virus isolation of the total number of pigs. Numbers in parentheses indicate virus titers presented as log_10_ mean ± SEM TCID_50_ per ml of BALF or g of tissue. Virus titers within rows with no common superscript letters differ significantly (P < 0.05) when compared by Welch's t-test. Values with the same superscript are not statistically different. Values in bold are statistically different from non-infected controls.

**Figure 2:**
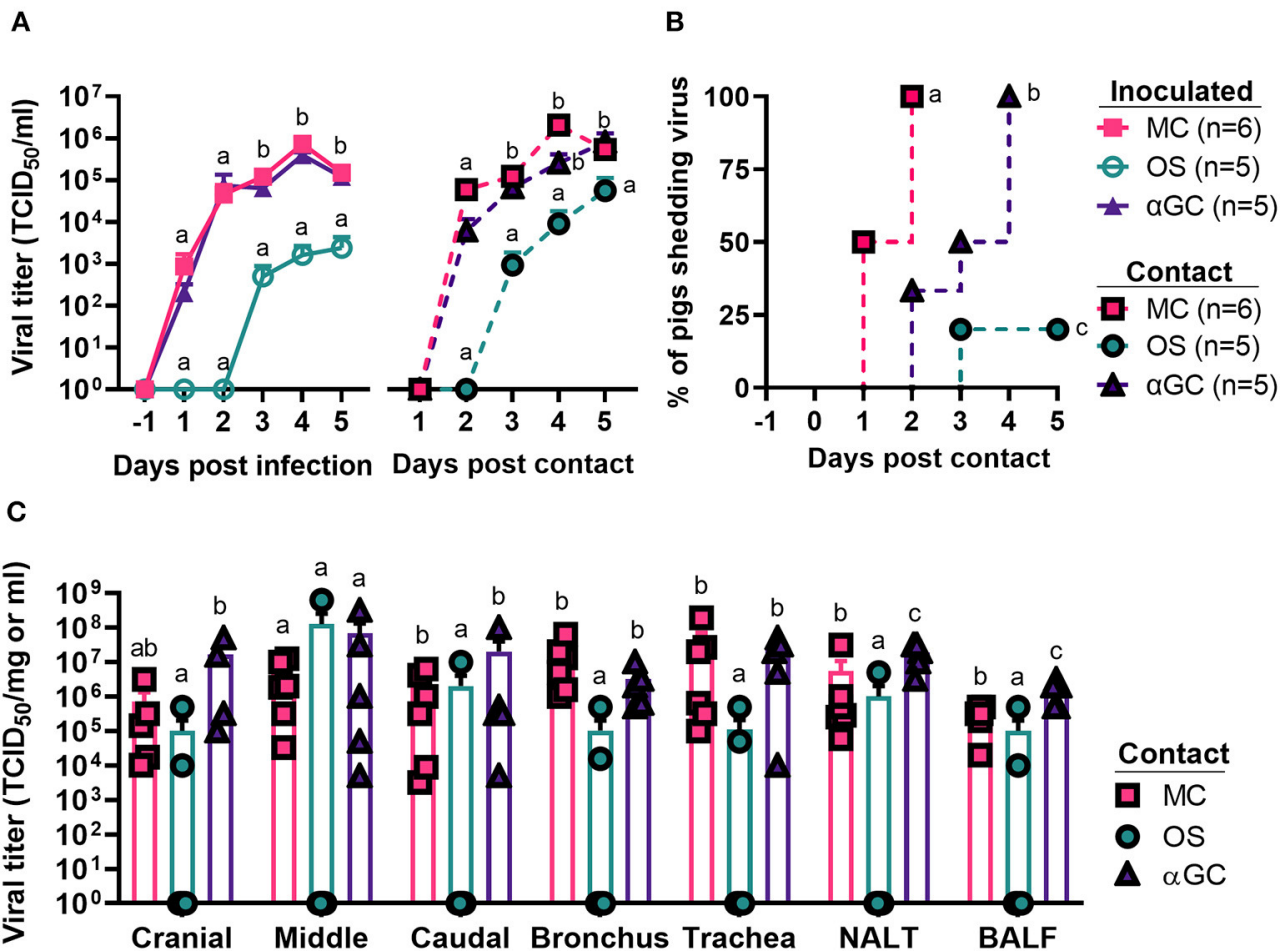
Viral load of contact pigs. **(A)** Virus titers in nasal swabs of principal inoculated and contact pigs during the 5-day post infection and -post contact periods, respectively. Data are represented as mean ± SEM. Values within days with no common superscript letters differ significantly (*P* < 0.05) when analyzed using Tukey's test. Values with the same superscript are not statistically different. **(B)** Incidence of contact pigs shedding virus after exposure to principal infected pigs. Survival curves with no common superscript letters differ significantly (*P* < 0.05) when compared by the log-rank test. **(C)** Virus titers for homogenized airway tissues and bronchoalveolar lavage fluid (BALF) collected from contact pigs at 5 d.p.c. Data are represented as mean ± SEM. Virus titers within tissues with no common superscript letters differ significantly (*P* < 0.05) when compared by Welch's *t*-test. Values with the same superscript are not statistically different. Symbols represent individual pigs.

In our second study which measured virus transmission, we detected virus shedding in 6/6, 5/5, and 1/5 contact pigs co-housed with MC, αGC, and OS pigs, respectively (Figures 2A,B). All contact pigs co-housed with MC and αGC pigs shed virus by day 4 post-contact. The single influenza positive OS contact pig began shedding low levels of virus at 3 d.p.c. Analysis of the BioSamplers^®^ devices detected 4.53 x 10^2^ infectious virions in 720 liters of air collected from the room housing MC pigs. In contrast, no infectious virus was detected in the room housing OS pigs. Analysis of the respiratory tract samples collected at 5 d.p.c. detected virus in 6/6, 5/5, and 2/5 contact pigs that were, respectively co-housed with MC, αGC, and OS pigs. OS contact pigs had lower average virus titers in cranial and caudal lung, bronchi, trachea, nasal turbinates, and BALF compared to MC and αGC contact pigs (Figure 2C). These combined results indicate that oseltamivir treatment reduced viral replication in the respiratory tract, which decreased virus transmission. In contrast, α-GalCer had no significant impact on virus replication or shedding.

### Lung pathology

**Figure 3:**
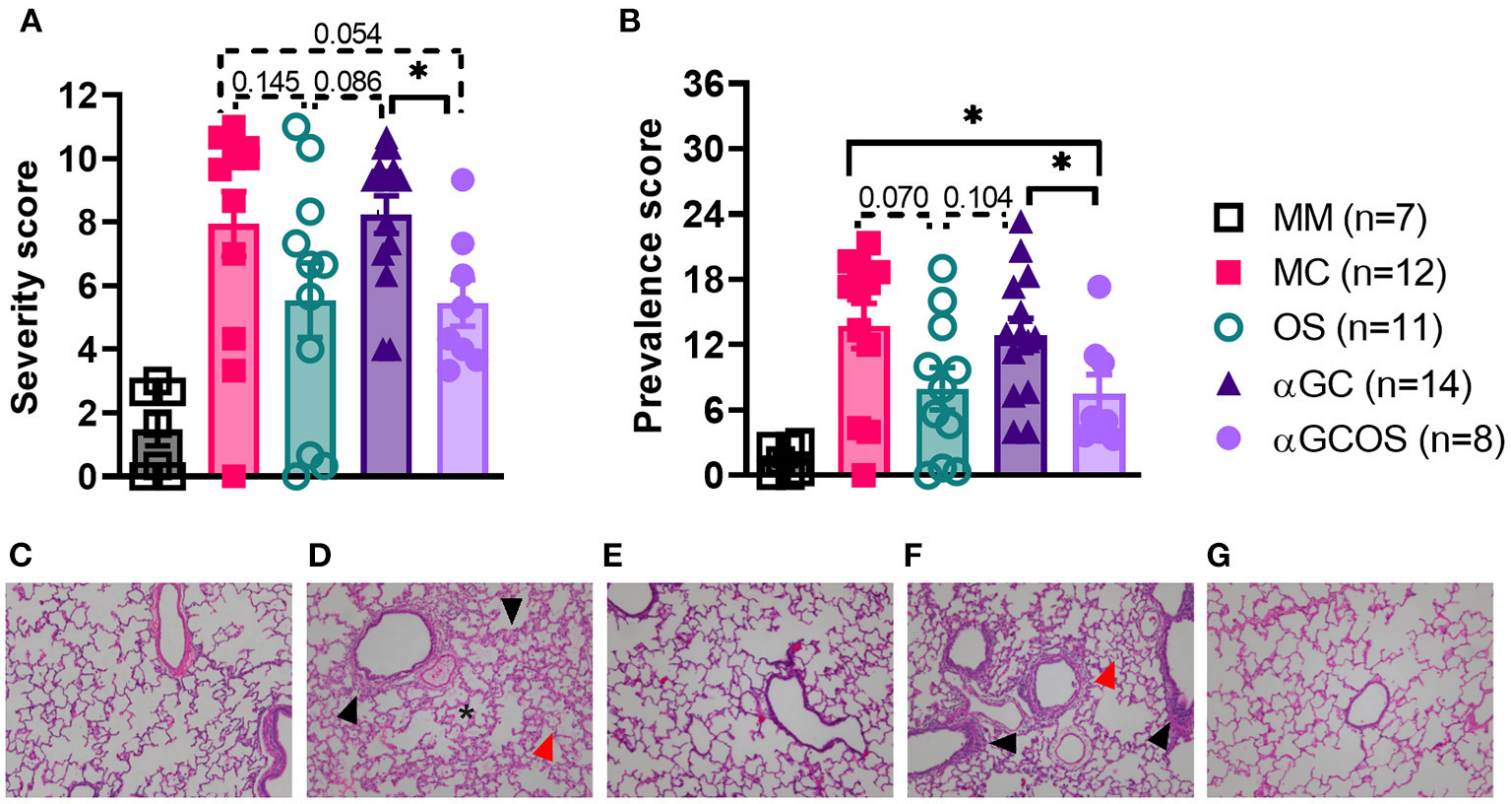
Overall severity and prevalence of lung pathology. Lung tissue from the right middle lung lobe was microscopically examined to determine the impact of oseltamivir and α-GalCer on influenza-induced immunopathology. Lung lesions were scored for the size and distribution of immune cell infiltrates, as described in section Lung immunopathology. **(A)** Pneumonia, bronchitis, and bronchiolitis scores were summed to calculate an overall severity score out of 12. **(B)** An overall disease prevalence score was calculated by multiplying the overall severity score by the prevalence score, with a maximum possible score of 36. Differences in severity and prevalence scores were compared by the Mann-Whitney test. Data are represented as mean ± SEM. *P < 0.05. Actual *P* values provided when 0.2 > *P* value > 0.05. Symbols represent individual pigs. **(C–G)** Representative transverse sections of lung tissue for the indicated treatment groups stained with H&E at 10x magnification. **(C)**: MM, **(D)**: MC, **(E)**: OS, **(F)**: αGC, and **(G)**: αGCOS. In MC and αGC groups there was mild thickening of alveolar septa by type II pneumocyte hyperplasia in peribronchiolar areas (black arrows) with small to moderate leukocyte septal infiltration, presence of pulmonary alveolar proteinosis (asterisk), and increased numbers of alveolar macrophages (red arrows).

Lung lesions were scored for the size and distribution of immune cell infiltrates, as described in section Materials and methods. Oseltamivir monotherapy, respectively reduced the severity and prevalence of pneumonia by 45 and 56 percent compared to MC pigs (Figure 3). OS pigs also tended to have less prevalent and less severe bronchitis and bronchiolitis compared to MC pigs. α-GalCer monotherapy did not significantly reduce lung pathology. Furthermore, combining α-GalCer and oseltamivir did not significantly reduce lung pathology compared to oseltamivir treatment alone. A comparison of the combined disease severity and prevalence scores found that OS and αGCOS pigs had fewer lesions compared to MC and αGC pigs (Figure 3).

### Flow cytometric analysis of leukocytes

Flow cytometry was used to assess immune cell populations in the lung, BALF and spleen. No differences were detected in the frequency of total lymphocytes, DCs, or macrophages between treatments for any of these tissues (Supplementary Figure 1, Supplemental Tables 2–4). However, the αGCOS group had a higher percentage of NK cells in BALF and spleen while OS pigs had a higher percentage of NK cells in their lungs. iNKT-cell frequencies tended to be higher in the spleens of αGC and αGCOS pigs compared to the other groups. We also observed that combination therapy resulted in lower percentage of γδ T cells in the lung and higher concentrations of CD8 cytotoxic T-cells (CD8αβ^+^CD4^−^) in the spleen.

### Gene expression

**Figure 4:**
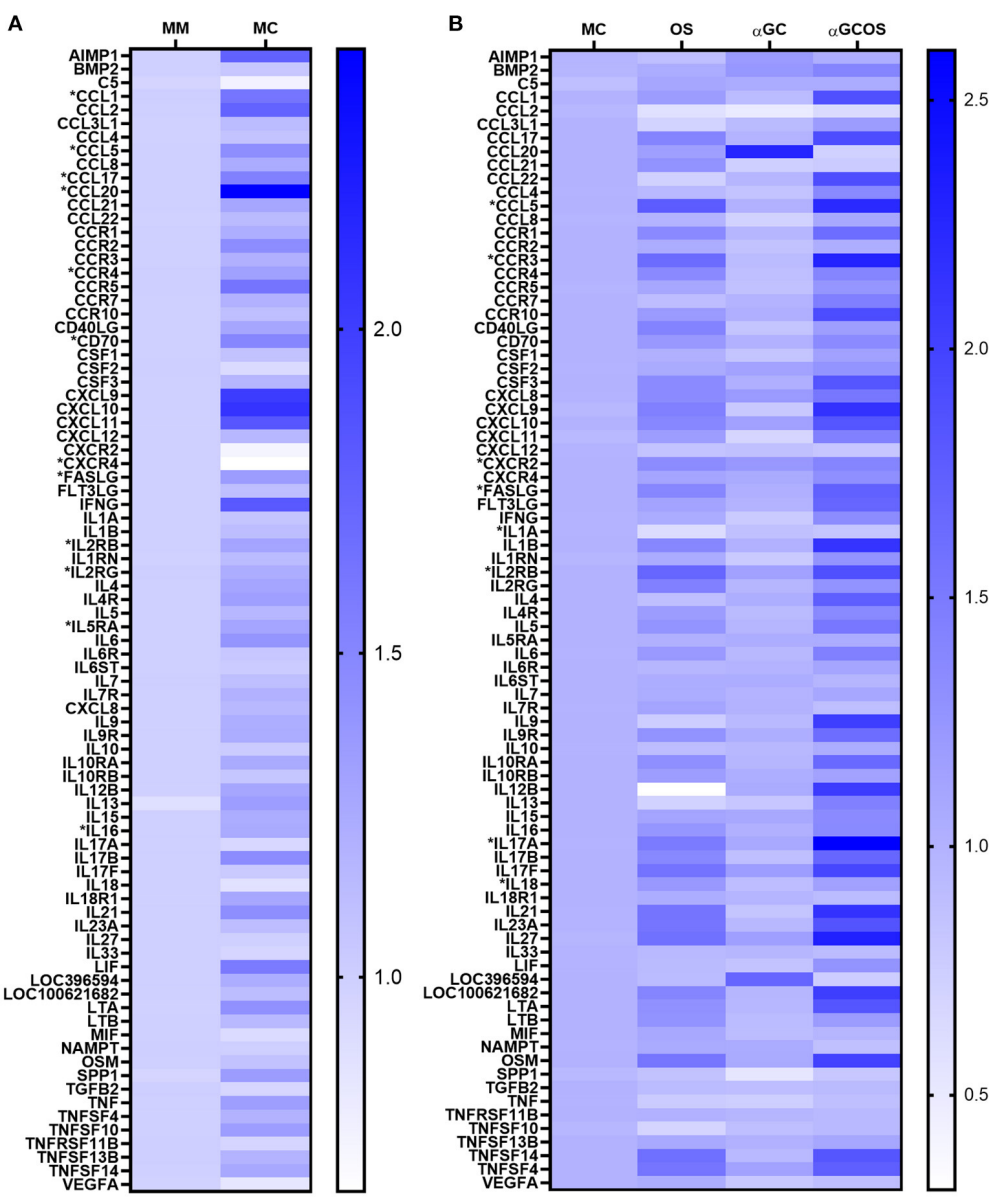
Inflammatory gene expression profile of lung tissue collected at 5 d.p.i. Transcript levels of 84 inflammatory genes were measured in combined samples from the left cranial, middle, and caudal lung lobes of 3 MM, 6 MC, 4 OS, 6 αGC, and 4 αGCOS pigs. **(A)** Heat map representing the relative value of MC gene expression normalized to MM pigs. **(B)** Heat map representing the relative value of OS, αGC, and αGCOS gene expression normalized to MC pigs. Genes statistically different between treatments are preceded by *. See Supplemental Table 5 for a complete list of fold-change values.

An RT-qPCR array was used to compare transcript levels of 84 pro-inflammatory markers in lung tissue collected at 5 d.p.i. Twelve genes were differentially expressed in MC compared to MM pigs (Figure 4A, Supplemental Table 5), including 5 chemokine ligands (*CCL1, CCL5, CCL17, CCL20, FASLG*), 5 chemokine/cytokine receptors (*CCR4, CXCR4, IL2RB, IL2RG, IL5RA*), the cytokine *IL16*, and the T-cell costimulatory molecule *CD70*. A KEGG pathway enrichment analysis found several of these genes were associated with cell survival (*IL2RB, IL2RG, IL4, IL5RA, FASLG*) and anti-influenza immune responses (*FASLG, CCL5, CXCL10*). To determine the effect of oseltamivir and α-GalCer on influenza-induced gene expression changes, we compared the OS, αGC, and αGCOS groups to the MC group (Figure 4B, Supplemental Table 5). The only gene differentially expressed between αGC and MC pigs was *CCL20*, which encodes a small cytokine important for the trafficking of innate immune cells into the lung (26–28). Overall, OS and αGCOS pigs displayed a similar pattern of gene expression changes compared to the MC group. However, OS pigs expressed less *IL1A* and more *CXCR2* and *IL18* compared to MC pigs while αGCOS pigs upregulated *CCL5, CCR3, IL17A, FASLG*, and *IL2RB*. A KEGG analysis of genes that were differentially expressed between MC and αGCOS pigs at the *p* < 0.1 level found enrichment of pathways involved in T helper 17 cell differentiation (*IL1B, IL2RB, IL2RG, IL4, IL4R, IL17A, IL17F, IL21, IL23A, IL27*) and anti-influenza immune responses (*FASL, IL1, IL1B, IL12, CCL5*).

## Discussion

Here, we used the swine influenza challenge model to compare a single intranasal administration of α-GalCer to a 5-day course of oseltamivir for reducing the severity and transmissibility of an IAV infection. We also investigated whether combining α-GalCer and OS would act synergistically to mitigate the infection. Pigs were administered 100 μg/kg α-GalCer since a previous study found that this dose inhibited virus replication in swine (23). Oseltamivir was administered at 150 mg/day, the recommended dose for adult/adolescent humans (29).

Oseltamivir reduced virus titers in most parts of the airway, but especially the upper respiratory tract. A similar outcome was observed in oseltamivir-treated ferrets challenged with an H5N1 IAV (30). Other studies have reported inconsistent results. Sidwell et al. (31) measured a 2-to-4-fold reduction in virus titers in the lungs of BALB/c mice administered 10 mg/kg/day of oseltamivir after infection with a lethal dose of the H1N1 strain A/NWS/33. However, the same regimen failed to inhibit the replication of two H3N2 viruses in mice. In another study, oseltamivir either significantly reduced or had no effect on virus titers of ferrets intranasally infected with a low (1 x 10^2^ PFU) and high (1 x 10^6^ PFU) dose of H1N1pdm09, respectively (32). Moreover, Govorkova et al. reported lower virus titers in the lung but not in nasal washes of oseltamivir-treated ferrets infected with H1N1pdm09 (33). Evidence supporting that oseltamivir inhibits virus replication in humans includes several reports that oseltamivir-treated influenza patients had lower virus concentrations in nasal and throat swabs compared to untreated patients (11, 34–36). A previous report which tested oseltamivir in pigs found only a modest impact on virus replication. This study differed from our own in that (i) it used 11-week-old pigs, (ii) virus inoculations were administered i.n., (iii) it used swine IAVs instead of H1N1pdm09, and (iv) oseltamivir treatment was initiated 12 h prior to infection (29).

One of our most significant findings is that oseltamivir prevented 3 of 5 contact pigs from contracting influenza, despite sharing the same pen with primary inoculated pigs during the most infectious period of disease (2–5 d.p.i.). To our knowledge, only a few ferret studies have examined the impact of oseltamivir on virus transmission (37–39). Two of these studies found that treating H1N1pdm09-infected ferrets 24 or 36 h after infection had no effect on virus transmission to contact ferrets (38, 39). However, a third study reported that administering oseltamivir to ferrets starting at 2 h after infection with human IAVs, including H1N1pdm09, significantly reduced secondary infections (37). This protocol was similar to our study in regards to the short interval between infection and treatment.

Oseltamivir clearly reduced influenza-induced lung lesions. This is notable since evidence supporting that oseltamivir reduces lung immunopathology is somewhat controversial (35, 40). It was shown that influenza-infected mice and ferrets administered oseltamivir prophylactically or on the day of infection had significantly less lung consolidation compared to untreated controls (32, 33, 41). However, treating cynomolgus macaques with oseltamivir did not reduce influenza-associated acute lung injury compared to placebo-treated animals (42). There is limited evidence that oseltamivir reduces pneumonia in humans since influenza patients are not usually subjected to radiological examination. However, a meta-analysis of 20 randomized, placebo-controlled trials of adults and children with influenza infections found that oseltamivir had no effect on reducing hospitalizations from lower respiratory tract complications (6). In contrast, a report describing nine randomized trials, including some overlapping with the previous citation, found that oseltamivir decreased the severity of lower respiratory tract complications (43).

α-GalCer had little impact on virus shedding, virus replication, or lung immunopathology. This contrasts with previous mouse studies that showed substantially reduced virus titers and lung pathology after α-GalCer administration (16–18). The discrepancy may be due to a variety of factors, including that iNKT-cells are much less frequent in pigs than in most inbred mouse strains. Another factor is that significant differences exist in mouse and porcine antiviral immune defenses, which may affect how iNKT-cells contribute to host-pathogen interactions and to the antiviral defense system (44–46). In contrast to the current study, we previously reported that i.n. administration of 100 μg/kg α-GalCer significantly reduced virus titers and lung pathology in pigs of similar breed and age to the current work (23). This discrepancy suggests that the outcome of α-GalCer therapy in swine is unpredictable and probably depends on a variety of host and/or environmental factors. These likely include iNKT-cell frequency and/or effector functions which vary considerably among pigs and is largely controlled by genetic factors (47). Since pigs within each study were closely related, one explanation for our results may be that pigs in the previous and present studies expressed iNKT cells that were genetically similar in their proclivity to stimulate strong and weak antiviral immune responses, respectively.

In conclusion, our study demonstrated that administering oseltamivir to influenza-infected pigs significantly reduced viral replication and lung immunopathology in principal animals and virus transmission to contact pigs. These findings support the use of NAIs during influenza outbreaks, especially for reducing onward transmission of infections. In contrast, α-GalCer did not alter the course of disease, even when used in combination with oseltamivir. Since swine resemble humans for iNKT-cells and are considered a reliable translational model for human influenza infections, our work suggests that iNKT-cell therapy may not be a viable approach for treating humans with influenza virus infections.

## Data availability statement

The original contributions presented in the study are included in the article/Supplementary material, further inquiries can be directed to the corresponding author.

## Ethics statement

The animal study was reviewed and approved by the institutional animal care and use committee at the University of Florida.

## Author contributions

DM, JR, and JD: conceived and designed study. DM, WG, BA, GY, JL, and JAL: collected complied and analyzed data. WC and IH: pathology analyses. DM: statistical analyses. DM, JL, and JD: drafted and edited manuscript. All authors contributed to the article and approved the submitted version.

## Funding

This research was funded jointly by the U.S. Department of Agriculture Grant 2021-67015 (JD) and the National Institutes of Health Grant HD092286 (JD and JR). It was also partially funded by the AMP and MCB Cores of the Center of Emerging and Zoonotic Infectious Diseases (CEZID) from the National Institute of General Medical Sciences (NIGMS) under award number P20GM130448 (JR), the NIAID Centers of Excellence for Influenza Research and Surveillance under contract number HHSN 272201400006C (JR), and the NIAID supported Center of Excellence for Influenza Research and Response (CEIRR, Contract Number 75N93021C00016 to JR).

## Conflict of interest

The authors declare that the research was conducted in the absence of any commercial or financial relationships that could be construed as a potential conflict of interest.

## Publisher's note

All claims expressed in this article are solely those of the authors and do not necessarily represent those of their affiliated organizations, or those of the publisher, the editors and the reviewers. Any product that may be evaluated in this article, or claim that may be made by its manufacturer, is not guaranteed or endorsed by the publisher.
