# Supplementary material for: Comparison of oseltamivir and α-galactosylceramide for reducing disease and transmission in pigs infected with 2009 H1N1 pandemic influenza virus
Source: Front Vet Sci. 2022 Oct 20;9:999507. doi: 10.3389/fvets.2022.999507 (PMC9635317; doi:10.3389/fvets.2022.999507)
Supplement: Supplementary file 1 [file Data_Sheet_1.pdf]

Supplementary Material

Supplementary Figure 1

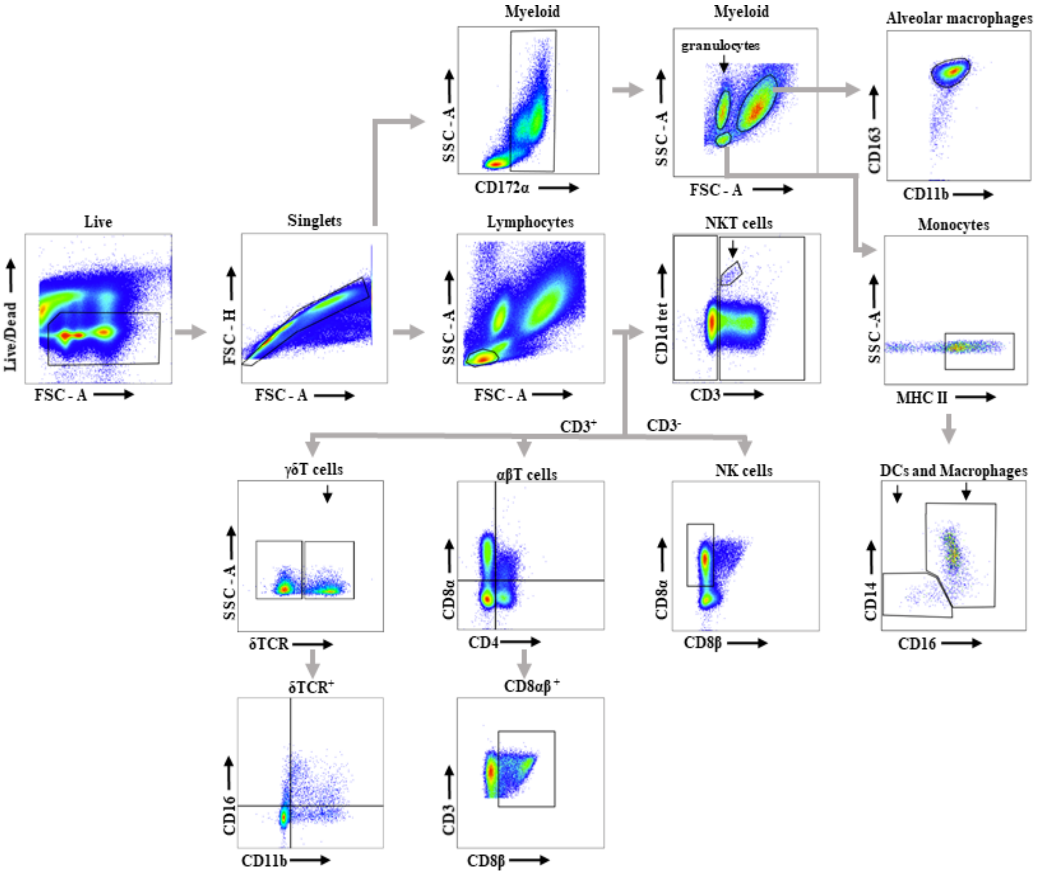

Supplemental Figure 1. Gating strategy to distinguish immune cell populations in tissues. αβ and γδ T cells were distinguished by sequentially gating live single lymphocytes, $CD3^+$ cells, and then $CD4^+$, $CD8\alpha^+$, $CD8\beta^+$, TCR-δ^+ cells. iNKT-cells were distinguished from conventional T cells according to CD1d-tetramer staining. NK cells were identified as $CD8\alpha^+$ $CD3^-$ cells after first gating on single live lymphocytes. T cell and NK cell subsets were distinguished according to their surface expression of CD11b and CD16. Monocytes, macrophages, and granulocytes were identified after initially gating on live single $CD172\alpha^+$ cells. Thereafter, monocytes were identified as $MHCII^+$ $CD14^+$ cells and alveolar macrophages were identified as SCC^{high} $CD163^+$ $CD11b^+$ cells. Granulocytes were identified according to FSC and SSC. FSC, forward scatter; SSC, side scatter; A, signal area; H, signal height. One representative lung sample is shown.

Supplemental Table 1. Reagents used for flow cytometry analysis of surface markers

Antigen	Clone	Isotype	Conjugation	Source
CD1d tetramer	N/A	N/A	PE	NIH Tetramer Core
Live/Dead	N/A	N/A	IR	Invitrogen
Anti-CD3ε	BB23-8E6-8C8	Mouse IgG2a κ	PE-Cy7	BD Biosciences
Anti-CD4	74-12-4	Mouse IgG2b κ	PE & Alexa647	Southern Biotech
Anti-CD8α	76-2-11	Mouse IgG2a κ	Alexa488 & Biotin	Southern Biotech
Anti-CD8*β*	PPT23	Mouse IgG1	A488	Bio-Rad
Anti-TCRδ	PGBL22A	Mouse IgG1	Alexa647	WSU mAB Center
Anti-CD14	MIL2	Mouse IgG2b	Alexa488	Bio-Rad
Anti-CD16	G7	Mouse IgG1	Biotin	BD Biosciences
Anti-CD11b	M1/70	Rat IgG2b κ	Alexa647	Biolegend
Anti-MHC class II	H42A	Mouse IgG2a κ	Alexa647	WSU mAb Center
Anti-CD163	2A10/11	Mouse IgG1	PE	Bio-Rad
Anti-CD172α	74-22-15A	Mouse IgG2b κ	PerCP	BD Biosciences

Supplemental Table 2. Flow cytometric analysis of immune cell populations in lung

	MM (n=6)	MC (n=10)	OS (n=8)	αGC (n=10)	αGCOS (n=8)
Lymphocytes (% of live cells)	26.6 ± 4.5[a]	28.5 ± 3.8[a]	29.3 ± 5.0[a]	26.6 ± 2.7[a]	25.8 ± 2.3[a]
Myeloid cells (% of live cells)	70.1 ± 3.3[a]	65.8 ± 2.9[ab]	57.1 ± 4.7[ab]	**63.0 ± 1.7[b]**	67.5 ± 3.2[ab]
iNKT-cells (% of $CD3^+$)	0.31±0.13[ab]	0.13±0.03[a]	0.08±0.02[a]	0.40±0.19[ab]	0.31±0.16[a]
$CD8\alpha^+CD8\beta^+CD4^-$ (% of $CD3^+$)	16.7 ± 5.8[a]	17.6 ± 4.7[a]	26.4 ± 10.8[a]	15.0 ± 3.2[a]	20.3 ± 6.2[a]
$CD8\alpha^-CD4^+$ (% of $CD3^+$)	8.15 ± 0.9[a]	7.76 ± 1.0[a]	6.09 ± 2.1[a]	9.39 ± 1.4[a]	7.69 ± 0.9[a]
δTCR^+ (% of $CD3^+$)	53.0 ± 8.7[a]	50.1 ± 7.0[a]	48.6 ± 6.4[a]	48.3 ± 5.3[a]	**30.1 ± 3.3[b]**
NK cells (% of live cells)	7.8 ± 1.8[a]	7.2 ± 1.1[a]	**8.7 ± 1.9[b]**	7.9 ± 1.2[a]	6.8 ± 0.9[a]
Alveolar macrophages (% of live cells)	36.2 ± 2.3[a]	38.5 ± 3.2[a]	32.8 ± 1.9[a]	38.1 ± 1.4[a]	40.6 ± 1.7[a]
Granulocytes (% of live cells)	18.6 ± 2.8[a]	14.3 ± 1.8[a]	20.7 ± 4.4[a]	16.7 ± 2.1[a]	19.0 ± 2.0[a]

Values expressed in means and ± SEM, P<0.05 calculated using R function glm followed by Tukey's test. Values within rows with no common superscript letters differ significantly. Values with the same superscript are not statistically different. Values in bold are statistically different from non-infected controls.

Supplemental Table 3. Flow cytometric analysis of immune cell populations in BALF

	MM (n=6)	MC (n=10)	OS (n=8)	αGC (n=10)	αGCOS (n=8)
Lymphocytes (% of live cells)	4.2 ± 0.9[a]	6.3 ± 1.6[a]	7.0 ± 1.1[a]	6.1 ±0.9[a]	7.1 ± 1.5[a]
Myeloid cells (% of live cells)	91.7 ± 0.8[a]	88.7 ± 1.5[a]	88.9 ± 1.7[a]	89.2 ± 1.2[a]	90.2 ± 1.7[a]
iNKT-cells (% of $CD3^+$)	0.57 ± 0.26[a]	0.37 ± 0.12[a]	0.13 ± 0.05[a]	0.36 ± 0.12[a]	0.57 ± 0.25[a]
$CD8\alpha^+CD8\beta^+CD4^-$ (% of $CD3^+$)	14.2 ± 3.3[ab]	14.6 ± 2.6[a]	14.4 ± 2.6[ab]	20.5 ± 4.9[ab]	**19.5 ± 3.5[b]**
$CD8\alpha^-CD4^+$ (% of $CD3^+$)	4.7 ± 1.5[a]	7.9 ± 2.0[a]	3.7 ± 1.2[a]	7.9 ± 1.7[a]	5.8 ± 2.0[a]
δTCR^+ (% of $CD3^+$)	40.8 ± 3.4[a]	37.5 ± 4.0[a]	38.6 ± 4.3[a]	44.0 ± 5.7[a]	34.4 ± 3.3[a]
NK cells (% of live cells)	5.0 ± 1.7[a]	3.9 ± 0.7[a]	4.4 ± 1.1[a]	5.3 ± 1.3[a]	**8.0 ± 1.9[b]**
Alveolar macrophages (% of live cells)	60.9 ± 4.4[a]	65.8 ± 5.8[a]	64.4 ± 2.4[a]	66.7 ± 2.4[a]	62.8 ± 3.7[a]
Granulocytes (% of live cells)	15.9 ± 3.3[a]	11.2 ± 3.1[a]	10.4 ± 1.6[a]	11.1 ± 1.2[a]	12.7 ± 1.8[a]

Values expressed in means and ± SEM, P<0.05 calculated using R function glm followed by Tukey's test. Values within rows with no common superscript letters differ significantly. Values with the same superscript are not statistically different. Values in bold are statistically different from non-infected controls.

Supplemental Table 4. Flow cytometric analysis of immune cell populations in spleen

	MM (n=6)	MC (n=10)	OS (n=8)	αGC (n=10)	αGCOS (n=8)
Lymphocytes (% of live cells)	65.9 ± 3.6[a]	63.9 ± 1.2[a]	60.2 ± 1.5[a]	61.5 ± 1.7[a]	59.7 ± 1.5[a]
Myeloid cells (% of live cells)	25.5 ± 2.5[a]	22.0 ± 1.6[a]	27.7 ± 1.6[a]	25.0 ± 1.8[a]	26.7 ± 2.7[a]
iNKT-cells (% of $CD3^+$)	0.06 ± 0.03[a]	0.08 ± 0.01[a]	0.10 ± 0.02[a]	0.36 ± 0.23[a]	0.29 ± 0.21[a]
$CD8\alpha^+CD8\beta^+CD4^-$ (% of $CD3^+$)	6.8 ± 0.7[a]	7.7 ± 0.8[a]	10.8 ± 1.4[a]	7.6 ± 0.8[a]	**12.5 ± 4.0[b]**
$CD8\alpha^-CD4^+$ (% of $CD3^+$)	22.3 ± 1.2[a]	19.5 ± 1.4[a]	21.2 ± 2.0[a]	17.30 ± 2.3[a]	14.0 ± 2.2[b]
δTCR^+ (% of $CD3^+$)	38.0 ± 3.9[a]	34.5 ± 2.2[a]	35.2 ± 3.9[a]	30.8 ± 2.1[a]	28.1 ± 2.9[a]
NK cells (% of live cells)	3.7 ± 0.6[a]	4.3 ± 1.0[a]	4.9 ± 2.0[ab]	3.7 ± 1.0[a]	**5.9 ± 2.5[b]**
Granulocytes (% of live cells)	10.2 ± 1.0[a]	7.5 ± 0.9[a]	9.4 ± 1.7[a]	9.1 ± 0.9[a]	10.2 ± 1.5[a]
Macrophages (% of live cells)	3.3 ± 0.6[a]	2.7 ± 0.3[a]	2.6 ± 0.3[a]	2.0 ± 0.2[a]	3.1 ± 0.6[a]
Dendritic cells (% of live cells)	1.3 ± 0.2[a]	1.9 ± 0.6[a]	2.1 ± 0.7[a]	2.0 ± 0.5[a]	1.1 ± 0.4[a]

Values expressed in means and ± SEM, P<0.05 calculated using R function glm followed by Tukey's test. Values within rows with no common superscript letters differ significantly. Values with the same superscript are not statistically different. Values in bold are statistically different from non-infected controls.

Supplemental Table 5. List of 84 transcripts queried through Pig Inflammatory Cytokines and Receptors PCR arrays in lung samples

	Normalized to MM		Normalized to MC					
	MC		OS		αGC		αGCOS	
**Gene**	**Fold change**	**P value**	**Fold change**	**P value**	**Fold change**	**P value**	**Fold change**	**P value**
AIMP1	-1.16	0.4503	1.09	0.590	1.05	0.714	1.09	0.641
BMP2	1.04	0.7640	1.32	0.261	1.09	0.357	1.49	0.087
C5	-1.27	0.7127	1.25	0.674	1.21	0.648	1.19	0.721
CCL1	1.62	**0.0057**	1.20	0.361	-1.08	0.911	1.88	0.086
CCL17	1.53	**0.0129**	1.42	0.282	-1.01	0.893	1.90	0.094
CCL2	1.75	0.3182	-1.63	0.411	-1.93	0.128	-1.49	0.319
CCL20	2.43	**0.0007**	1.17	0.305	2.27	**0.011**	-1.39	0.386
CCL21	1.28	0.1543	1.29	0.334	-1.32	0.130	-1.28	0.104
CCL22	1.16	0.5154	-1.38	0.455	-1.04	0.877	1.90	0.094
CCL3L1	1.16	0.7266	-1.42	0.172	-1.07	0.454	1.20	0.214
CCL4	1.10	0.7576	-1.06	0.776	-1.16	0.421	1.38	0.178
CCL5	1.45	**0.0270**	1.78	0.143	1.01	0.724	2.23	**0.005**
CCL8	1.24	0.4638	1.01	0.952	-1.37	0.238	1.11	0.949
CCR1	1.23	0.2664	1.37	0.292	-1.03	0.848	1.63	0.106
CCR10	1.12	0.5358	1.21	0.363	1.03	0.684	1.92	0.081
CCR2	1.45	0.0738	1.06	0.734	-1.17	0.149	1.04	0.901
CCR3	1.22	0.2986	1.63	0.217	-1.09	0.289	2.28	**0.029**
CCR4	1.32	**0.0177**	1.36	0.262	-1.12	0.278	1.40	0.179
CCR5	1.63	0.1202	1.12	0.783	-1.14	0.464	1.27	0.393
CCR7	1.21	0.2722	-1.10	0.534	-1.01	0.877	1.46	0.137
CD40LG	1.28	0.2005	1.43	0.135	-1.19	0.265	1.19	0.371
CD70	1.51	**0.0382**	1.24	0.279	1.02	0.981	1.35	0.156
CSF1	1.09	0.4686	1.02	0.810	-1.19	0.344	1.15	0.244
CSF2	-1.09	0.9730	1.08	0.979	1.16	0.674	1.28	0.512
CSF3	1.18	0.3153	1.35	0.301	1.03	0.660	1.83	0.084
CXCL10	4.00	0.4869	-1.39	0.492	-1.67	0.351	-1.06	0.499
CXCL11	1.85	0.3866	1.26	0.597	-1.37	0.355	1.53	0.524
CXCL12	1.17	0.3228	-1.18	0.432	-1.14	0.303	-1.21	0.196
LOC396594	1.24	0.2751	-1.08	0.833	1.70	**0.030**	-1.32	0.079
CXCL9	3.10	0.3803	1.00	0.813	-1.80	0.339	1.48	0.956
CXCR2	-1.34	0.0713	1.34	**0.048**	1.24	0.060	1.40	0.088
CXCR4	-1.49	**0.0267**	1.13	0.465	1.08	0.598	1.32	0.076
FASLG	1.36	**0.0436**	1.39	0.221	1.03	0.775	1.73	**0.033**
FLT3LG	1.12	0.4384	1.14	0.400	-1.00	0.947	1.68	0.106
IFNG	1.82	0.1129	1.08	0.727	-1.26	0.284	1.36	0.295

Cont. Supplemental Table 5. List of 84 transcripts queried through Pig Inflammatory Cytokines and Receptors PCR arrays in lung samples

	Normalized to MM		Normalized to MC					
	MC		OS		αGC		αGCOS	
**Gene**	**Fold change**	**P value**	**Fold change**	**P value**	**Fold change**	**P value**	**Fold change**	**P value**
IL10	1.03	0.8229	-1.1	0.578	-1.04	0.710	1.06	0.885
IL10RA	1.26	0.0826	1.31	0.315	-1.04	0.795	1.65	0.097
IL10RB	1.06	0.5386	1.19	0.206	1.03	0.697	1.14	0.287
IL12B	1.29	0.1875	-1.50	0.434	1.06	0.549	2.06	0.074
IL13	1.52	0.4106	1.62	0.287	-1.22	0.378	1.44	0.298
IL15	1.24	0.1116	1.13	0.435	1.10	0.359	1.35	0.087
IL16	1.25	**0.0056**	1.25	0.288	1.02	0.686	1.36	0.112
IL17A	-1.05	0.9814	1.51	0.310	1.09	0.967	2.63	**0.045**
IL17F	1.03	0.8871	1.58	0.256	1.19	0.257	1.98	0.080
IL18	-1.15	0.2879	1.23	0.096	-1.11	0.334	1.15	0.276
IL18R1	1.28	0.1945	1.05	0.623	-1.02	0.935	-1.09	0.322
IL1A	1.08	0.6679	-1.61	0.084	-1.13	0.336	-1.21	0.321
IL1B	1.12	0.5335	1.39	0.301	1.01	0.912	2.14	0.097
IL1RN	1.15	0.6687	1.10	0.732	-1.23	0.316	1.31	0.538
IL21	1.45	0.3383	1.57	0.262	-1.18	0.427	2.17	0.063
IL23A	1.12	0.5668	1.56	0.248	-1.05	0.750	1.85	0.070
IL27	-1.00	0.7592	1.63	0.271	1.21	0.614	2.37	0.078
IL2RG	1.23	**0.0091**	1.45	0.247	-1.02	0.642	1.28	0.067
IL4	1.29	0.0579	-1.13	0.441	1.04	0.660	1.74	0.093
IL4R	1.33	0.0851	1.20	0.329	-1.07	0.581	1.37	0.090
IL5	1.17	0.2299	1.27	0.323	-1.03	0.741	1.53	0.128
IL5RA	1.30	**0.0147**	1.02	0.584	1.05	0.476	1.05	0.441
IL6	1.41	0.3889	1.24	0.426	-1.04	0.643	1.47	0.311
IL6R	1.05	0.5318	-1.04	0.831	-1.01	0.983	1.11	0.240
IL6ST	1.03	0.7342	1.05	0.667	1.05	0.624	-1.03	0.699
IL7	1.11	0.2073	1.06	0.580	-1.00	0.981	1.09	0.389
IL7R	1.21	0.4104	1.13	0.642	1.01	0.993	-1.12	0.711
CXCL8	1.16	0.4022	1.36	0.291	1.23	0.104	1.54	0.140
IL9	1.24	0.4571	1.39	0.303	-1.06	0.583	2.05	0.091
LIF	1.58	0.1535	-1.07	0.747	-1.16	0.651	1.28	0.339
OSM	1.09	0.5958	1.54	0.248	1.07	0.452	2.01	0.069
IL17B	1.47	0.0768	1.38	0.276	-1.13	0.855	1.68	0.107
IL33	-1.04	0.9174	-1.05	0.891	-1.03	0.929	-1.09	0.528
TNFSF14	1.28	0.2285	1.61	0.222	-1.06	0.940	1.83	0.123
IL9R	1.24	0.2142	1.29	0.323	1.04	0.712	1.62	0.131

Cont. Supplemental Table 5. List of 84 transcripts queried through Pig Inflammatory Cytokines and Receptors PCR arrays in lung samples

	Normalized to MM		Normalized to MC					
	MC		OS		αGC		αGCOS	
**Gene**	**Fold change**	**P value**	**Fold change**	**P value**	**Fold change**	**P value**	**Fold change**	**P value**
LOC100 621682	1.14	0.5615	1.41	0.290	-1.04	0.870	2.05	0.097
IL2RB	1.3	**0.0443**	1.68	0.156	1.16	0.124	1.88	**0.042**
LTA	1.44	0.1861	1.30	0.326	-1.04	0.886	1.83	0.126
LTB	1.15	0.2934	1.28	0.322	-1.09	0.701	1.20	0.323
MIF	-1.09	0.2081	1.09	0.365	-1.10	0.096	-1.04	0.559
NAMPT	-1.00	0.9252	1.08	0.698	1.07	0.634	-1.14	0.380
SPP1	1.41	0.5498	-1.09	0.892	-1.76	0.160	-1.16	0.497
TGFB2	-1.06	0.3733	-1.08	0.344	-1.11	0.326	-1.09	0.178
TNF	1.34	0.1863	-1.29	0.399	-1.34	0.336	-1.13	0.778
TNFRSF 11B	-1.05	0.7505	1.01	0.892	-1.02	0.943	-1.07	0.576
TNFSF1 0	1.36	0.3374	-1.37	0.344	-1.06	0.569	-1.01	0.762
TNFSF1 3B	1.19	0.0975	1.08	0.419	1.01	0.890	1.09	0.391
TNFSF4	1.21	0.4030	1.55	0.258	1.15	0.250	1.75	0.116
